# Effect of Nano-Sized Heat Transfer Enhancers on PCM-Based Heat Sink Performance at Various Heat Loads

**DOI:** 10.3390/nano10010017

**Published:** 2019-12-19

**Authors:** Nadezhda S. Bondareva, Mikhail A. Sheremet

**Affiliations:** Laboratory on Convective Heat and Mass Transfer, Tomsk State University, Tomsk 634050, Russia; bondarevans@mail.tsu.ru

**Keywords:** nanoparticles, phase change material, natural convection, heat-generating element

## Abstract

Many passive heat controlling technologies are based on the use of phase change materials. As a rule, at low operation temperatures, close to environmental conditions, paraffins or fatty acids with melting points of 20–90 °C are used. However, the low thermal conductivity of these materials requires the development of various heat transfer enhancers satisfying technical requirements. In this work, the possibility of nanoparticle application to the heat transfer augmentation inside a closed copper radiator filled with pure n-octadecane, depending on the thermal conditions of the local heater and other system parameters, are numerically investigated.

## 1. Introduction

The creation of effective cooling systems is caused by the development of electronic technology and mobile devices. With a decrease in the size of functional elements, productivity and, as a result, the density of heat fluxes in small volumes are increased. Active cooling systems limited by space requirements cannot cope with intense heat generation under conditions of unsteady peak loads [1,2]. In such conditions, conventional metal radiators quickly overheat, requiring intensive forced convective cooling. One of the developing types of heat sinks is heat sinks based on PCM (phase change material) [1,3,4], whose effective heat capacity is increased due to the latent energy of phase transitions. Paraffins or fatty acids with melting points corresponding to operating conditions are used here as materials. The only drawback of these materials is their low thermal conductivity (0.14–0.5 W/(m·K)) [5], due to which, in consequence, they are not used in their pure form. Such a material can be used in combination with various heat transfer enhancers, mainly using high-conductivity metal radiators with immersed metal foams, matrixes, or the addition of highly heat-conducting particles [3,6,7,8,9].

Thermophysical properties of materials can be changed strongly with the addition of nano-sized particles. These properties depend on the nanoparticle’s concentration [9,10,11,12,13]. When studying heat and mass transfer in nano-enhanced phase change materials (NePCMs) or nanoliquids, changes in nanoparticle concentration should be taken into account, as well as its effect on dynamic and thermal properties. Currently, there are many investigations devoted to the stability of NePCMs [12,14,15,16,17,18,19]. In addition, there are various surfactants for nanoparticles ensuring the stability of suspensions for a sufficiently long time [7,13,20].

It is known that the addition of nanoparticles raises the thermal conductivity of the material [7,13,20]. Thermophysical properties of n-octadecane with the addition of TiO_2_ nanoparticles were experimentally measured by Motahar et al. [21]. The experiments were carried out both in liquid and solid state for the temperature range between 5 °C and 55 °C; the mass concentration of particles in this case was varied from 0% to 5%. It was shown that thermal conductivity can be increased with a growth of the nanoparticle’s concentration in both solid and liquid phases. However, the maximum thermal conductivity was observed for 3% at all considered sample temperatures. Nourani et al. [13] showed a monotonic increase in the thermal conductivity of paraffin with alumina nanoparticles. The concentration of nanoparticles in paraffin with a melting point of 54 °C–58 °C ranged between 0% and 10%. With a rise of mass concentration up to 10%, the melting time can be reduced by 27% and, at the same time, the effective heat capacity of the material is decreased.

An NePCM-based heat sink with a heat pipe was examined by Kumar et al. [22]. It was shown that the use of a heat sink significantly reduced the temperature in the system, and the addition of NePCM contributed to a longer charging process and an increase in temperature going much slower.

The ability of PCMs to accumulate and release energy when a certain temperature is reached to change properties under various thermal influences can be used in many engineering fields; for example, in latent heat thermal storages, constructions, solar collectors, or metasurfaces [6,7,23,24,25,26,27,28,29,30,31,32]. Using PCM can reduce daily room temperature fluctuations, reduce air conditioning costs at high heat exposure, and solar radiation. The addition of nanoparticles makes it possible to accelerate the phase transition processes within the structure by increasing thermal conductivity and heat capacity [25,26]. Khan et al. [7] analyzed the effect of different types of nanoparticles on heat transfer in a horizontal multitube system. The two-dimensional problem of natural convection and melting in a closed circular region filled with RT44HC with a melting point of 41 °C–44 °C and *L_m_* = 255 kJ/kg was considered, and several energy sources were placed inside the region. The effect of fourteen types of nanoparticles of metal oxides on the melting rate was analyzed, where the effect of the nanoparticles’ concentration on the thermal conductivity, viscosity, and latent heat of NePCM was taken into account. The authors showed that SiO_2_, Al_2_O_3_, MgO, and TiO_2_ turned out to be the most effective nano-additives, which showed the highest melting rates in combination with a slight decrease in latent heat, and these materials were the most economically advantageous in comparison with the other oxides considered.

An experimental study [6] focused on the use of RT35HC with Al_2_O_3_ nanoparticles to lower the temperature of the photovoltaic system. The NePCM–filled system with internal pin-finning was heated by the element with constant heat generation. It was shown that the presence of internal pin-fins significantly reduces the panel temperature; at the same time, adding 0.77% of nanoparticles reduces the surface temperature by 4.5%. It was also shown that the use of an NePCM-based heat sink diminishes the panel temperature by 36.9–52.3% at various thermal loads. An experimental study of the properties of paraffin with Si_3_N_4_ nanoparticles was carried out by Yang et al. [20]. It was shown that the addition of nanoparticles to the material causes an increase in thermal conductivity by 35%. Moreover, it was found that the latent energy increases by 3.4% with a low concentration of nanoparticles (at about 1%), and further growth of concentration leads to a reduction of latent heat in consequence of a decrease in the volume fraction of base material.

It should be noted that the effect of nanoparticles on melt viscosity is essential [11,21,30]. The addition of nanoparticles changes the dynamic properties of the material. Namely, along with an increase in thermal conductivity, a significant increase in the viscosity of the melt is observed, which leads to changes in the hydrodynamics of the melt flow. Ho and Gao [11] studied the properties of n-octadecane combined with a low concentration of Al_2_O_3_ nanoparticles. It has been experimentally shown that a decrease in viscosity plays a large role in the melting process. With an increase in the nanoparticles’ volume fraction, the thermal conductivity and viscosity are raised. Thus, the interaction of these two effects does not lead to an intensification of the melting process.

The problem of melting of RT44 with Al_2_O_3_ or CuO nanoparticles inside an aluminum heat sink at a constant heat flux was considered by Bayat et al. [33]. An increase in the mass fraction of alumina nanoparticles by 4% leads to a slight reduction in the temperature within the heat source; however, when raising the particle’s concentration to 6%, the effect weakens, which is associated with a significant increase in the viscosity of the melt. After that, the paraffin has completely melted and the lowest temperature is observed in the case of pure paraffin.

In the present study, the two-dimensional problem of paraffin melting inside a closed finned heat sink in the presence of a volumetric heat source was considered. A model describing the heat and mass transfer inside a paraffin cavity was formulated using the non-primitive variables such as the stream function (u=∂ψ/∂y, v=−∂ψ/∂x) and vorticity (ω=∂v/∂x−∂u/∂y), and this boundary value problem was solved using the finite difference method. To formulate and solve the energy equation between the melt and the solid material, a porous layer was introduced with a smoother transition of the thermophysical properties and the enthalpy function. Hydrodynamic equations were solved in the melt region using the Boussinesq approximation. At the boundaries between the materials, the conditions for the equality of heat fluxes and temperature were set. The Biot criterion was used to describe the heat exchange with an environment. The solution of the unsteady problem allows obtaining the local characteristics of melt flow and heat transfer within the considered system and the dependences of the heat transfer rate and the average heater temperature with time. Based on the data obtained, a detailed analysis of the influence of the nanoparticles’ concentration on the heat transfer regimes during unsteady heat generation was carried out.

## 2. Mathematical Model

**Figure 1:**
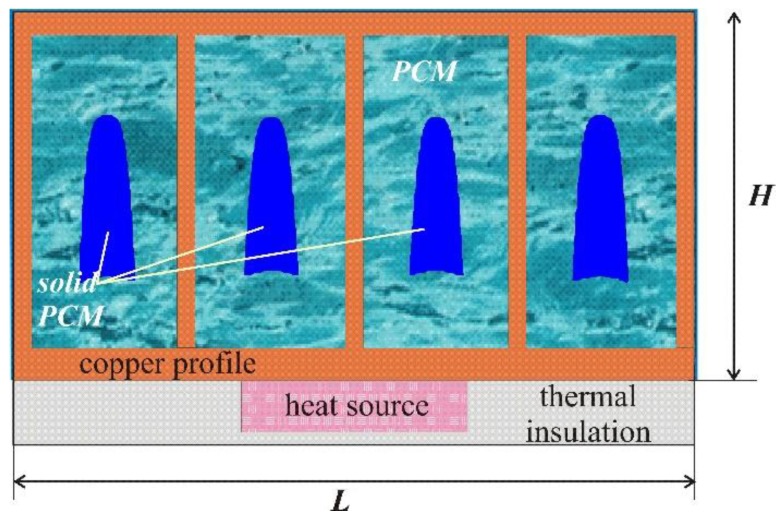
Domain of interest.

The natural convective melting of a nano-enhanced phase change material in a cavity with a heat sink under the effect of a heat-generating local element was studied. In this study, the cavity had a rectangular shape with aspect ratios *L*:*H* = 2. The radiator size (*L* × *H*) was 3 cm × 1.5 cm. Figure 1 presents a scheme of the region under consideration including a closed metal radiator filled with pure n-octadecane, under which a source with unsteady volumetric heat generation is located. The conjugate natural convection problem is considered taking into account the phase transitions in paraffin, whose melting point is 301.05 K. It was believed that at the initial time, the radiator and heat source were evenly cooled by ambient temperature (in this study, *T*_0_ = 296 K). Heat exchange with the external environment was carried out using the Newtonian cooling conditions that were set at the upper and lateral boundaries of the profile, while the remaining boundaries of the system were thermally insulated. It was assumed that the melt of paraffin with nanoparticles is a viscous incompressible fluid, and the flow arising in the melt region is laminar. Material properties were considered to be temperature independent and constant within a single phase. To describe the properties of a material suspended by nanoparticles, a single-phase nanofluid model was used, which assumes a uniform distribution of particles over the volume [34,35,36].

The Boussinesq approximation was used to describe the buoyancy force influence within the Navier–Stokes equations. The mass conservation equation and momentum equations for describing the mass transfer inside the melt region are as follows [35]:

(1) $$\frac{\partial u}{\partial x}+\frac{\partial v}{\partial y}=0$$

(2) $${\left(\rho_{l}\right)}_{nm}\left(\frac{\partial u}{\partial t}+u\frac{\partial u}{\partial x}+v\frac{\partial u}{\partial y}\right)=-\frac{\partial p}{\partial x}+{\left(\mu_{l}\right)}_{nm}\left(\frac{\partial^{2}u}{\partial x^{2}}+\frac{\partial^{2}u}{\partial y^{2}}\right)$$

(3) $${\left(\rho_{l}\right)}_{nm}\left(\frac{\partial v}{\partial t}+u\frac{\partial v}{\partial x}+v\frac{\partial v}{\partial y}\right)=-\frac{\partial p}{\partial y}+{\left(\mu_{l}\right)}_{nm}\left(\frac{\partial^{2}v}{\partial x^{2}}+\frac{\partial^{2}v}{\partial y^{2}}\right)+{\left(\rho_{l}\beta_{l}\right)}_{nm}g\left(T-T_{m}\right)$$

Heat transfer in solid medium was carried out by conductive heat transfer. The heat conduction equations were considered for the heat source, a metal radiator, and paraffin wax. The energy conservation equation for the local heater was written taking into account the time-dependent volumetric heat generation expressed through the harmonic law *Q* = *Q*_0_ (1 − sin(2π*bt*)). Heat transfer inside the PCM was carried out due to the conductive and convective regimes. In addition to heat transfer inside the solid and liquid regions, it is necessary to take into account the production and absorption of latent heat at the moving boundary of the phase transition; therefore, the energy equations for the solid and liquid phases are formulated in variables of enthalpy and temperature as follows [35,37]:

(4) $$\frac{\partial h}{\partial t}={\left(k_{s}\right)}_{nm}\left(\frac{\partial^{2}T}{\partial x^{2}}+\frac{\partial^{2}T}{\partial y^{2}}\right)$$

(5) $$\frac{\partial h}{\partial t}+u\frac{\partial h}{\partial x}+v\frac{\partial h}{\partial y}={\left(k_{l}\right)}_{nm}\left(\frac{\partial^{2}T}{\partial x^{2}}+\frac{\partial^{2}T}{\partial y^{2}}\right)$$

At the same time, at the phase change border, the Stefan condition is realized as a boundary condition, which takes into account the conversion of latent heat into sensible heat and the movement of the boundary in space: [k∂T∂n¯]=−LmVn, where on the left side one can find the heat flux in the direction of the normal vector $\bar{n}$ and on the right side we have the multiplication of the specific latent energy and the velocity of the phase change line in the direction along the normal vector *V_n_*.

The system of unsteady differential Equations (1)–(5) combined with the equations of heat conduction for a radiator and heat generation element, previously reduced to the dimensionless form, were solved using the non-primitive variables such as stream function and vorticity. The following values were chosen as the scales for time, length, and other parameters:

$$\begin{array}{c} X=x/H,\;Y=y/H,\;U=u/\sqrt{g\beta_{l}\Delta TH},\;V=v/\sqrt{g\beta_{l}\Delta TH},\;\tau =t\sqrt{g\beta_{l}\Delta T/H},\\ f=b/\sqrt{g\beta_{l}\Delta T/H},\;\Theta =\left(T-T_{m}\right)/\Delta T,\;\Psi =\psi /\sqrt{g\beta_{l}\Delta TH^{3}},\;\Omega =\omega H/\sqrt{g\beta_{l}\Delta TH} \end{array}$$

In addition, to simplify the algorithm for solving the energy equations inside a PCM, auxiliary functions were introduced: *ϕ* (*T*), which characterizes the volume fraction of the melt, as well as *ξ*(*ϕ*) and *ζ*(*ϕ*) for a smooth transition of the thermal parameters of NePCM from one aggregate state to another one. As a result, Equations (4) and (5), together with the Stefan boundary condition, were transformed into the following equation [35,38,39]:

(6) $$\zeta \left(\phi \right)\left[\frac{\partial \Theta }{\partial \tau }+U\frac{\partial \Theta }{\partial X}+V\frac{\partial \Theta }{\partial Y}\right]+\frac{\rho_{nm}L_{nm}}{\rho_{l}L_{m}}\cdot Ste\cdot \left[\frac{\partial \phi }{\partial \tau }+U\frac{\partial \phi }{\partial X}+V\frac{\partial \phi }{\partial Y}\right]=\frac{\xi \left(\phi \right)}{\sqrt{Ra\cdot \mathrm{Pr}}}\left(\frac{\partial^{2}\Theta }{\partial X^{2}}+\frac{\partial^{2}\Theta }{\partial Y^{2}}\right).$$

Here, *ξ*(*ϕ*) and *ζ*(*ϕ*) characterize the changes in thermal conductivities and volumetric heat capacities, respectively:

$$\xi \left(\phi \right)=\frac{{\left(k_{s}\right)}_{nm}}{k_{l}}+\phi \left(\frac{{\left(k_{l}\right)}_{nm}}{k_{l}}-\frac{{\left(k_{s}\right)}_{nm}}{k_{l}}\right),\;\zeta \left(\phi \right)=\frac{{\left(\rho_{s}c_{s}\right)}_{nm}}{\rho_{l}c_{l}}+\phi \left(\frac{{\left(\rho_{l}c_{l}\right)}_{nm}}{\rho_{l}c_{l}}-\frac{{\left(\rho_{s}c_{s}\right)}_{nm}}{\rho_{l}c_{l}}\right)$$

The function *ϕ* (*T*) represents the following relationship:

$$\phi =\left\{ \begin{array}{ll} 0, & T<T_{m}-\eta \\ \frac{T-\left(T_{m}-\eta \right)}{2\eta }, & T_{m}-\eta \leq T\leq T_{m}+\eta \\ 1, & T>T_{m}+\eta \end{array}\right.$$

Here, *η* is the smoothing parameter and it equals to 0.005.

The heat conduction equations for a radiator and an element with volumetric heat generation, taking into account the accepted temperature scale, take the form:

(7) $$\frac{\partial \Theta }{\partial \tau }=\frac{\alpha_{1}/\alpha_{l}}{\sqrt{Ra\cdot \mathrm{Pr}}}\left(\frac{\partial^{2}\Theta }{\partial X^{2}}+\frac{\partial^{2}\Theta }{\partial Y^{2}}\right)$$

(8) $$\frac{\partial \Theta }{\partial \tau }=\frac{\alpha_{2}/\alpha_{l}}{\sqrt{Ra\cdot \mathrm{Pr}}}\left(\frac{\partial^{2}\Theta }{\partial X^{2}}+\frac{\partial^{2}\Theta }{\partial Y^{2}}+Os\left[1-sin\left(2\pi f\tau \right)\right]\right)$$

The melt flow equations using the non-primitive dimensionless variables take the form:

(9) $$\frac{\partial^{2}\Psi }{\partial X^{2}}+\frac{\partial^{2}\Psi }{\partial Y^{2}}=-\Omega$$

(10) $$\frac{\partial \Omega }{\partial \tau }+U\frac{\partial \Omega }{\partial X}+V\frac{\partial \Omega }{\partial Y}=\frac{\mu_{nm}/\mu_{l}}{{\left(\rho_{l}\right)}_{nm}/\rho_{l}}\sqrt{\frac{\mathrm{Pr}}{Ra}}\left(\frac{\partial^{2}\Omega }{\partial X^{2}}+\frac{\partial^{2}\Omega }{\partial Y^{2}}\right)+\frac{{\left(\rho_{l}\beta_{l}\right)}_{nm}/\left(\rho_{l}\beta_{l}\right)}{{\left(\rho_{l}\right)}_{nm}/\rho_{l}}\frac{\partial \Theta }{\partial X}$$

The following dimensionless complexes are involved in Equations (6)–(10):

- Rayleigh number—$Ra=g\beta_{l}\Delta TH^{3}/\left(\nu_{l}\alpha_{l}\right)$;
- Prandtl number—$Pr=\nu_{l}/\alpha_{l}$;
- Stefan number—$Ste=L_{m}/\left(c_{l}\Delta T\right)$, characterizing the latent heat effect;
- Ostrogradsky number—$Os=QH^{2}/\left(k_{2}\Delta T\right)$, characterizing the volumetric heat flux effect;
- Biot number—Bi=γH/k, characterizing the intensity of external cooling.

Equations (6)–(10) are closed by the initial and boundary conditions for the system under consideration, which can be written as follows:

- *τ* = 0: Θ = Θ*_out_*, ψ = 0, Ω = 0;
- *X* = 0 and *X* = 2, 0 ≤ *Y* ≤ 1: ${\frac{\partial \Theta }{\partial X}|}_{X=0}=Bi\left(\Theta -\Theta_{out}\right)$ and ${\frac{\partial \Theta }{\partial X}|}_{X=L/H}=-Bi\left(\Theta -\Theta_{out}\right)$;
- 0 ≤ *X* ≤ 0.6 and 1.4 ≤ *X* ≤ 2, *Y* = 0: ∂Θ/∂Y=0;
- 0.6 ≤ *X* ≤ 1.4, *Y* = 0: $\frac{\partial \Theta_{1}}{\partial Y}=\frac{k_{2}}{k_{1}}\frac{\partial \Theta_{2}}{\partial Y}$;
- 0 ≤ X ≤ 2, *Y* = 1: ${\frac{\partial \Theta }{\partial Y}|}_{Y=1}=-Bi\left(\Theta -\Theta_{out}\right)$;
- at the profile surface: $\frac{k_{nm}}{k_{1}}\frac{\partial \Theta_{nm}}{\partial \bar{n}}=\frac{\partial \Theta_{1}}{\partial \bar{n}}$;
- at the solid-liquid interface: Θ = 0;
- at *X* = 0.6 and *X* = 1.4, −0.2 ≤ *Y* ≤ 0: $\frac{\partial \Theta }{\partial X}=0$;
- at 0.6 ≤ *X* ≤ 1.4, *Y* = −0.2: $\frac{\partial \Theta }{\partial Y}=0$.

The outer temperature was set as Θ*_out_* = −0.0508, which corresponds to the dimensional temperature *T*_0_ = 296 K (the temperature scale in this study was equal to Δ*T* = 60 K), and the heat transfer coefficient at the upper and side boundaries was *γ* = 20 W/(m^2^K).

The boundary conditions for the velocity at all solid boundaries, including at the phase change line, were specified by the relations: ψ = 0, $\Omega =-\nabla^{2}\Psi$.

The numerical solution of partial differential equations was obtained on the basis of the finite difference method using the locally one-dimensional Samarsky scheme for the parabolic equations [35,36,39]. The algorithm developed was previously tested using the experimental data by Gau and Viskanta [40] and numerical data by Gong and Mujumdar [41].

**Figure 2:**
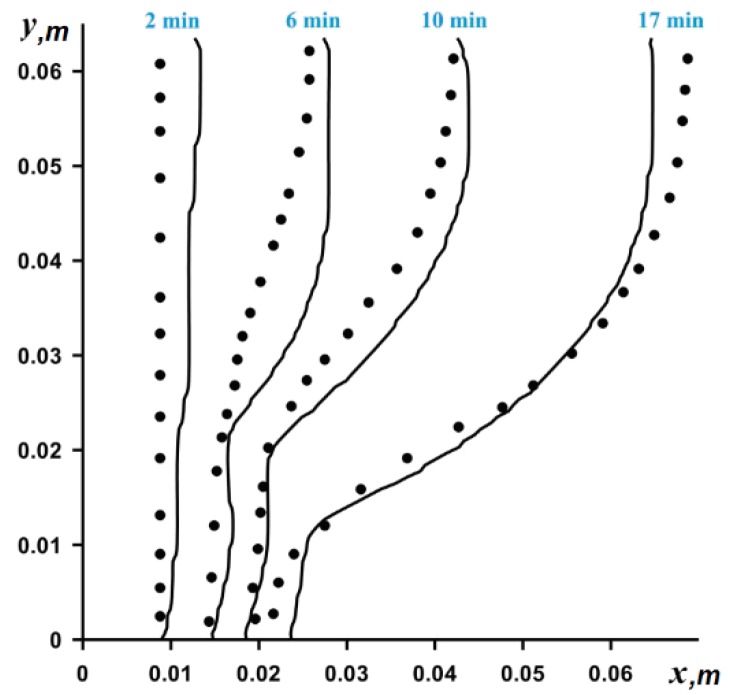
Comparison of phase change line position, dots (experimental data [40]) and solid lines (obtained numerical data).

Figure 2 shows a very good agreement for the phase change line in comparison with experimental data by Gau and Viskanta [40] for pure gallium melted in a differentially-heated rectangular cavity. The rectangular cavity filled with pure gallium in solid state begins to heat up from the side wall, the temperature of which is constant and above the melting point of gallium. A constant low temperature is maintained on the opposite vertical wall and the remaining boundaries are thermally insulated. Figure 2 shows the positions of the interphase boundary observed at different instants of time.

## 3. Thermophysical Properties of NePCM

In this work, a copper profile filled with n-octadecane and heated from a local heat source was considered. The material of the heat-generated element is corresponded to pure silicon. Table 1 presents the properties of the components of the considered system “heat sink—heat source”, on the basis of which calculations were carried out.

It was assumed that the nanoparticles were uniformly distributed over the volume occupied by paraffin, and the properties of the suspension were considered constant. The ratios for the thermophysical properties of NePCM depending on the mass concentration of particles are:

- Using the experimental data [43], a correlation for the thermal conductivity was obtained taking into account the Brownian diffusion influence:
  $$\begin{array}{l} {\left(k_{l}\right)}_{nm}=k_{l}\frac{k_{np}+2k_{l}-2\left(k_{l}-k_{np}\right)\Phi }{k_{np}+2k_{l}+\left(k_{l}-k_{np}\right)\Phi }+5\cdot 10^{4}\beta_{\lambda }\Phi \rho_{l}c_{l}\sqrt{\frac{\kappa T}{\rho_{np}d_{np}}}r\left(T,\Phi \right)\\ {\left(k_{s}\right)}_{nm}=k_{s}\frac{k_{np}+2k_{s}-2\left(k_{s}-k_{np}\right)\Phi }{k_{np}+2k_{s}+\left(k_{s}-k_{np}\right)\Phi } \end{array}$$
  In this relation, for thermal conductivity of NePCM, we have [43] $\beta_{\lambda }=8.4407{\left(100\Phi \right)}^{-1.07304}$ and $\kappa =1.381\cdot 10^{-23}J/K$ as the Boltzmann constant, while *r*(*T*,Φ) can be defined as:
  $$r\left(T,\Phi \right)=\left(2.817\cdot 10^{-2}\Phi +3.917\cdot 10^{-3}\right)\frac{T}{\tilde{T}}+\left(-3.0669\cdot 10^{-2}\Phi -3.91123\cdot 10^{-3}\right),\;\tilde{T}=273K$$
  The first term in correlation for (*k_l_*)*_nm_* and for (*k_s_*)*_nm_* is the Maxwell model. The second term was determined experimentally by Vajjha for liquids with the addition of Al_2_O_3_ nanoparticles at a concentration range Φ ≤ 10% and this term determines the Brownian motion of nanoparticles [43]. Since the particle motion also depends on the diameter of nanoparticles and temperature, the considered model includes the functions *r*(*T*,Φ) and *β_λ_*, which depend on these parameters.
- Dynamic viscosity [33]
  $$\mu_{nm}=0.983\cdot e^{12.959\Phi }\mu_{l}$$
- The coefficient of thermal volume expansion
  $${\left(\rho_{l}\beta_{l}\right)}_{nm}=\left(1-\Phi \right)\left(\rho_{l}\beta_{l}\right)+\Phi \left(\rho_{np}\beta_{np}\right)$$
- Volumetric heat capacity
  $$\begin{array}{l} {\left(\rho_{l}c_{l}\right)}_{nm}=\left(1-\Phi \right)\left(\rho_{l}c_{l}\right)+\Phi \left(\rho_{np}c_{np}\right)\\ {\left(\rho_{s}c_{s}\right)}_{nm}=\left(1-\Phi \right)\left(\rho_{s}c_{s}\right)+\Phi \left(\rho_{np}c_{np}\right) \end{array}$$
- Density
  $$\begin{array}{l} {\left(\rho_{l}\right)}_{nm}=\left(1-\Phi \right)\rho_{l}+\Phi \rho_{np}\\ {\left(\rho_{s}\right)}_{nm}=\left(1-\Phi \right)\rho_{s}+\Phi \rho_{np} \end{array}$$
- Heat capacity
  $$\begin{array}{l} {\left(c_{l}\right)}_{nm}={\left(\rho_{l}c_{l}\right)}_{nm}/{\left(\rho_{l}\right)}_{nm}\\ {\left(c_{s}\right)}_{nm}={\left(\rho_{s}c_{s}\right)}_{nm}/{\left(\rho_{s}\right)}_{nm} \end{array}$$
- Latent heat
  $$L_{nm}=\left(1-\Phi \right)\rho_{l}L_{m}/{\left(\rho_{l}\right)}_{nm}$$

The model for thermal conductivity and dynamic viscosity was carried out for aluminum oxide nanofluids in a wide range of temperatures and nanoparticle concentration (1% ≤ Φ ≤ 10% [34,43]) and tested in many numerical problems of melting and solidification [7,33,34].

## 4. Results and Discussion

Analysis was performed in a wide range of nanoparticle concentration and volumetric heat flux oscillation frequency.

**Figure 3:**
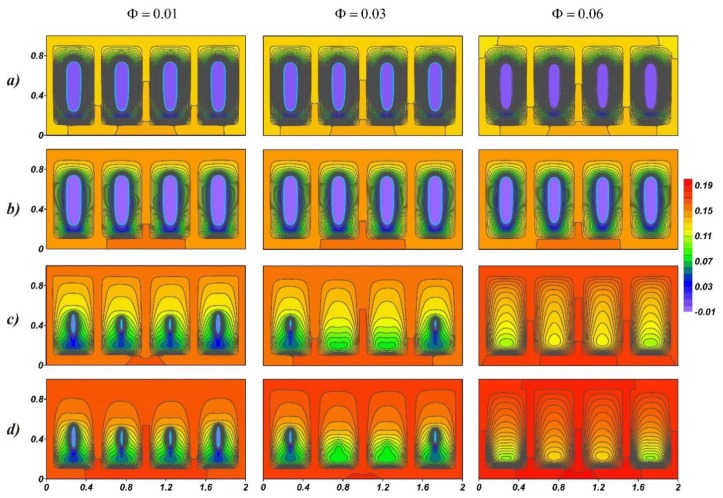
The temperature fields for *Os* = 0.0845, *τ* = 3200 (**a**,**b**), and *τ* = 4000 (**c**,**d**): (**a**,**c**) *f* = 200 and (**b**,**d**) *f* = 400.

Figure 3 shows temperature fields at small values of the Ostrogradsky number for different values of the nanoparticles’ concentration at time points 3200 and 4000, which correspond to the melting process of the material. At time τ = 3200, it can be seen that the temperature fields practically do not differ. The latter can be explained by high thermal conductivity and, as a result, the profile has the highest temperature. In paraffin, due to lower thermal conductivity, high-temperature gradients are observed on the surface of solid paraffin and the inner surface of the radiator. The melt located under the solid material region is cooled, which indicates a weak downward cooled convective flow. The melting rate changes with the increasing concentration of nanoparticles, namely due to an increase in thermal conductivity. The volume of solid material is lower at Φ = 0.06, which at time τ = 4000 reaches complete melting. The temperature in the profile is lower at *f* = 200.

**Figure 4:**
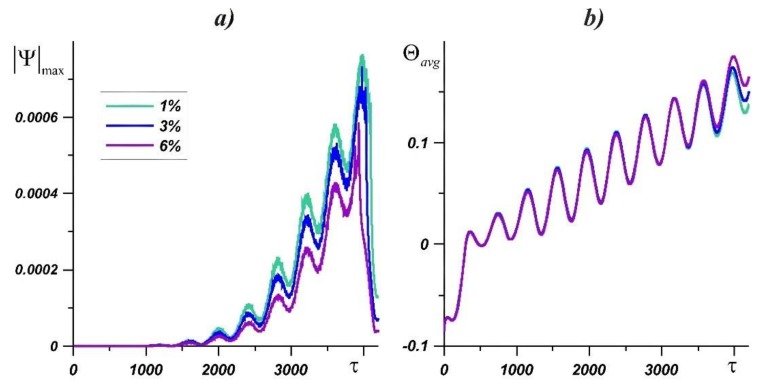
The maximum value of the stream function (**a**) and the average source temperature (**b**) in dependence on time for different nanoparticle concentrations and *Ra* = 4.03·10^6^, *Os* = 0.0845, *f* = 200.

Figure 4 presents the maximum value of the stream function and the average temperature in the heat source in dependence on time. Values of the ${\left|\Psi \right|}_{\max}$ increase with a rise of the source temperature and the melt region. It can be seen that the temperature in the source during the melting process is practically independent of the particle’s concentration, while the difference between the maximum values of the stream function increases. Due to an increase in melt viscosity, the natural convective heat transfer weakens; however, this effect compensates for a significant increase in thermal conductivity inside paraffin. At the same time, the lowest temperature in the profile is lower at Φ = 0.01. After the material has completely melted, the circulation of the melt sharply diminishes, while the temperature in the source rises sharply. After complete melting of the material, the effect of the concentration of nanoparticles appears at the source temperature. The source heats up more intensively with an increase in the concentration of nanoparticles. During the melting process, the local maxima of the temperature Θ*_avg_* do not differ significantly. After the paraffin completely melts, the lowest temperature in the source is observed at Φ = 0.01.

**Figure 5:**
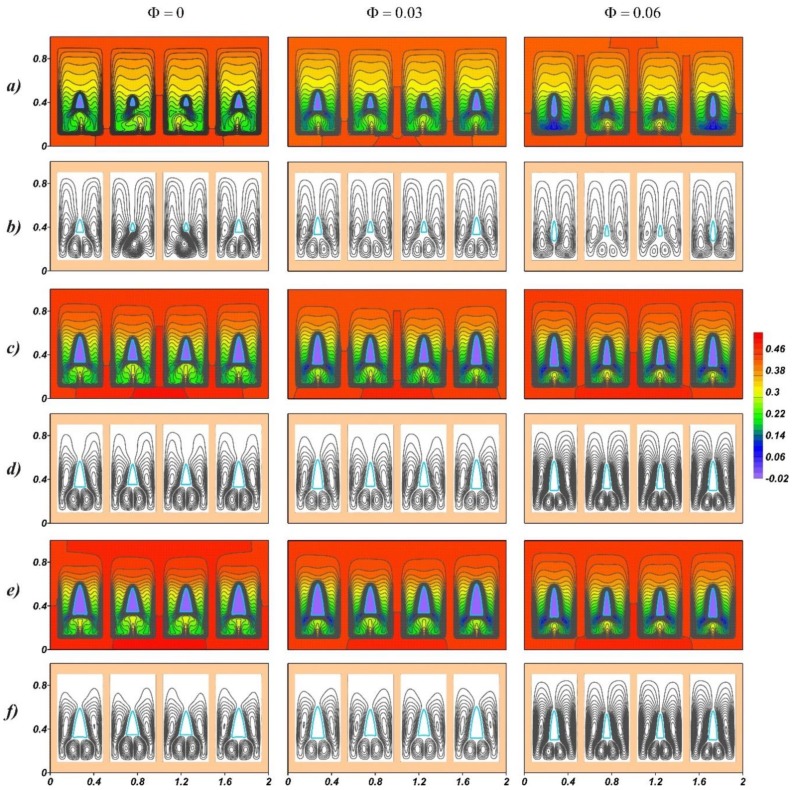
Temperature fields (**a**,**c**,**e**) and streamlines (**b**,**d**,**f**) for *Ra* = 9.55·10^6^, *Os* = 0.338, τ = 1600: (**a**,**b**) *f* = 200; (**c**,**d**) *f* = 400; (**e**,**f**) *f* = 800.

An increase in the Rayleigh number is associated with the dimensional scale of the system. Figure 5 shows the temperature fields and streamlines in the melt at *Ra* = 9.55·10^6^. Natural convection in this case develops much more intensively due to large volumes. It is seen that under the region of unmelted paraffin, additional vortices appear. Due to the appearance of additional convective cells in the upper part of the region, the melt also overheats. At high Rayleigh numbers, higher temperatures are observed at the same volume fraction of the melt. In the case of *f* = 400, the central temperature plumes are symmetrical, and the convective hydrodynamic structures are symmetrical, while the upper part of the melt region is warmer than in the case of *f* = 200 and the volume of solid paraffin is higher. If the frequency of power fluctuations increases, a higher volume of solid material is observed.

**Figure 6:**
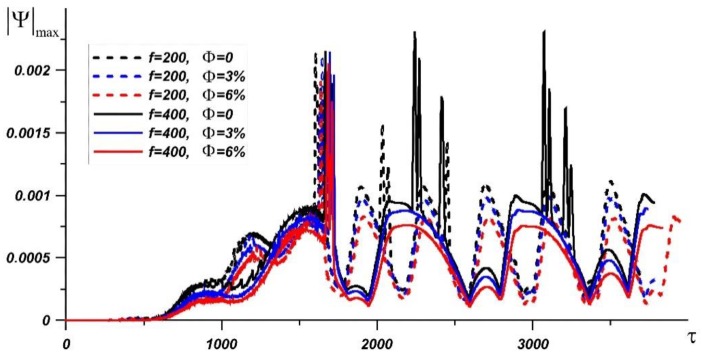
The maximum value of the stream function with time at *Ra* = 9.55·10^6^, *Os* = 0.338 for different *f* and nanoparticle volume fraction.

With increasing parameter *f*, the melting process begins later. From the graph of the dependence of the stream function maximum value, it is seen that the velocities inside the melt decrease with increasing *f*. Figure 6 shows ${\left|\Psi \right|}_{\max}$ values for different magnitudes of *f* and nanoparticle concentration. The intensity of natural convection is characterized by a high Rayleigh number, and an abrupt change in ${\left|\Psi \right|}_{\max}$ is observed in paraffin. After complete melting of paraffin in all cases considered, this jump is associated with the disappearance of the hard boundaries of paraffin, as a result of which the central ascending flows rise to the upper border, forming two global convective cells.

After the establishment of a convective regime in a region without phase transitions, sharp jumps in the stream function are observed only in pure paraffin during the maximum heat generation in the source. It should be noted that the temperature in the source first rises to the melting point, and then the graph goes to values close to zero before the melt region sufficient for the development of natural convection appears. The time period when the temperature in the source kept at the melting point of the material is associated with the small distance between the boundary of the phase transition and the walls of the radiator. Moreover, the concentration of nanoparticles does not significantly affect the temperature in the source. With the development of the natural circulation of the melt within the liquid region, the influence of the mass fraction of nanoparticles becomes noticeable. Thus, the temperature in the source at time moment τ = 1500 is 4% lower at Φ = 0.01, 2.8% at Φ = 0.03, and only 0.6% at Φ = 0.06 compared to pure paraffin, while the values of the stream function ${\left|\Psi \right|}_{\max}$ decrease by 0%, 9%, and 16.5%, respectively, at *f* = 400.

An important factor in determining the heat transfer regime is the position of the heating element. In the system under consideration, due to the high thermal conductivity of copper, the profile plays a decisive role in the energy dissipation in the system. The radiator warms up quickly and fairly evenly. In the case of a closed radiator, paraffin heats up from all closing boundaries. The formation and development of natural convection in this case is associated with a temperature gradient between the lower part of the profile and solid paraffin, while the heated melt is collected in the upper part of the region, reducing the heat exchange rate of paraffin with the radiator. As a result, the effect of convective heat transfer is much lower than in the case of an open radiator that does not have an upper wall, while the closed form of the radiator promotes an increase in conductive heat transfer in the system.

**Figure 7:**
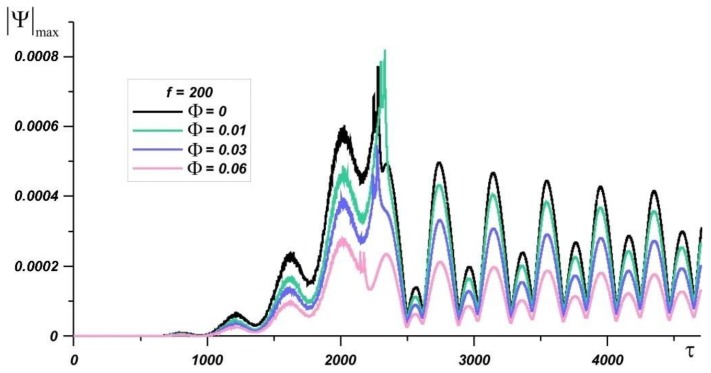
The maximum value of the stream function with time at *Ra* = 1.19·10^6^, *Os* = 0.0845, *f* = 200 for various nanoparticle concentrations.

A decrease in the Rayleigh number to 1.19·10^6^ leads to a decrease in the intensity of natural convection and hydrodynamic structures become more stable. It can be seen from Figure 7 that the presence of nanoparticles in the melt has a stronger effect on convective heat transfer in the melt. The relative reduction in the values of the stream function is much lower than in cases of *Ra* = 4.03·10^6^ and *Ra* = 9.55·10^6^. ${\left|\Psi \right|}_{\max}$ values with an addition of 1%, 3%, and 6% of the mass fraction of nanoparticles are decreased by 23%, 37%, and 55%, respectively, at time point τ = 2000 and by 14%, 33%, and 57% at time point τ = 3600 compared with pure paraffin. It is also worth noting that, at the same time, the temperature in the source decreases with the addition of nanoparticles.

**Figure 8:**
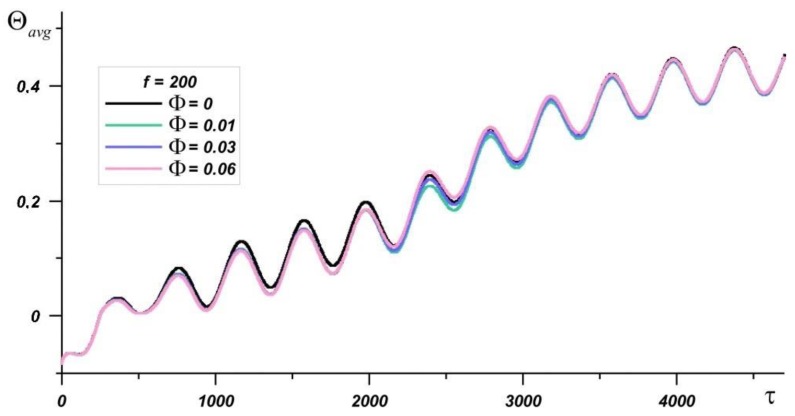
The average heater temperature with time at *Ra* = 1.19·10^6^, *Os* = 0.0845, *f* = 200 for various nanoparticle concentrations.

Figure 8 shows a graph of the evolution of the average source temperature at *Ra* = 1.19·10^6^, *f* = 200. It is seen that when solid paraffin predominates in the region, the addition of nanoparticles reduces the temperature; however, after the material is completely melted, the effect of nanoparticles on the source temperature decreases. So, at the time point τ = 3600, the minimum temperature is observed at Φ = 0.01 and the average temperature of the source is 1.5% lower than in pure paraffin. At time moment τ = 2000, the minimum average temperature in the source Θ*_avg_* is also observed at Φ = 0.01 and it is about 7% lower than in the case of pure paraffin application.

## 5. Conclusions

A numerical study of conjugate natural convection combined with melting process inside a closed copper radiator filled with paraffin with Al_2_O_3_ nanoparticles during unsteady heating was carried out. The influence of the system size and operation mode of the heater on the temperature distributions in the system and velocity fields in the melt at various concentrations of nanoparticles is analyzed. The average source temperature and maximum values of the stream function throughout the entire process were obtained.

It was shown that the effect of nanoparticles on system performance depends on the intensity of convective heat transfer. At high Rayleigh numbers (*f* = 400, *Ra* = 9.55·10^6^), the maximum values of the stream function decrease by 16.5% at a 6% concentration of nanoparticles, and the minimum average source temperature during the melting process is observed at 1% particle loadings. At lower Rayleigh numbers (*Ra* = 1.19·10^6^) in the case of *f* = 200 at peak loads, the decrease in ${\left|\Psi \right|}_{\max}$ reaches 57% compared to pure paraffin. Moreover, with a non-zero fraction of solid paraffin during high heat fluxes, the temperature of the source is 7% lower with the addition of 1% alumina nanoparticles. Higher concentrations considered (Φ = 0.03 and Φ = 0.06), an increase in viscosity weakens natural convective heat transfer and during melting does not have a greater effect. After the material has melted completely, the temperature of the source approaches the values obtained for pure paraffin. Thus, the addition of a small amount of nanoparticles allows the reduction of the source temperature, increasing the heat transfer rate inside the PCM; however, at higher nanoparticle concentrations (3% or more), an increase in the melt viscosity reduces the natural circulation caused by buoyancy forces, and an improvement of the thermal conductivity in the material does not compensate for this effect.

## Figures and Tables

**Table 1:** Properties of the materials.

Material		*k*, W/(m·K)	*c*, J/(kg·K)	*μ*, Pa·s	*ρ*, kg/m^3^	*β*, K^−1^
Paraffin, n-octadecane (*T_m_* = 301.05 K, *L_f_* = 2.41·10^5^ J/kg) [42]	solid	0.39	1900	–	814	8.5·10^−4^
	liquid	0.157	2200	3.8·10^−3^	770	
Aluminum oxide (nanoparticles) (*d* = 59·10^−9^ m) [33]		36	765	–	3600	7.8·10^−6^
Copper (radiator)		401	385	–	8900	–
Silicon (heat source)		148	714	–	2330	–

## Nomenclature

*b*	dimensional volumetric heat flux oscillation frequency, s^−1^
*Bi*	the Biot number
*c*	specific heat, JK^−1^ kg^−1^
*d*	diameter, m
*f*	non-dimensional volumetric heat flux oscillation frequency
*g*	gravitational acceleration, ms^−2^
*H*	cavity height, m
*h*	specific enthalpy, Jkg^−1^
*k*	thermal conductivity, WK^−1^m^−1^
*L*	cavity length, m
*L_m_*	fusion energy or latent heat of melting, Jkg^−1^
$\bar{n}$	normal to the surface
*Os*	Ostrogradsky number
*p*	pressure, Pa
*Pr*	Prandtl number
*Q*	heat transfer rate per unit of volume, W m^−3^
*Ra*	Rayleigh number
*Ste*	Stefan number
*t*	time, s
*T*	temperature, K
*T_m_*	melting temperature
*u*, *v*	velocity components in Cartesian coordinate along *x* and *y*, ms^−1^
*U*, *V*	dimensionless velocity components
*x*, *y*	Cartesian coordinate, m
*X*, *Y*	dimensionless Cartesian coordinates
**Greek symbols**	
*α*	thermal diffusivity, m^2^ s^−1^
*β*	coefficient of thermal expansion, K^−1^
*γ*	heat transfer coefficient, WK^−1^m^−2^
*η*	smoothing parameter (or melting temperature range), K
Θ	dimensionless temperature
*μ*	dynamic viscosity, Pa s
*ν*	kinematic viscosity, m^2^ s^−1^
*ρ*	density, kgm^−3^
*τ*	dimensionless time
Φ	nanoparticles volume fraction
*φ*	volume fraction of the melt
*ψ*	stream function, m^2^ s^−1^
Ψ	dimensionless stream function
*ω*	vorticity, s^−1^
Ω	dimensionless vorticity
**Subscripts**	
0	initial condition or ambient
1	radiator
2	heater
*l*	liquid
*m*	melting
*nm*	nanomaterial
*np*	nanoparticle
*s*	solid

## References

[1] Gharbi S., Harmand S., Jabrallah S.B. (2015). Experimental comparison between different configurations of PCM based heat sinks for cooling electronic components. Appl. Therm. Eng..

[2] Walsh E., Walsh P., Grimes R., Egan V. (2008). Thermal management of low profile electronic equipment using radial fans and heat sinks. J. Heat Transf..

[3] Baby R., Balaji C. (2013). Experimental investigations on thermal performance enhancement and effect of orientation on porous matrix filled PCM based heat sink. Int. Commun. Heat Mass Transf..

[4] Li Z.-W., Lv L.-C., Li J. (2016). Combination of heat storage and thermal spreading for high power portable electronics cooling. Int. J. Heat Mass Transf..

[5] Khan Z., Khan Z., Ghafoor A. (2016). A review of performance enhancement of PCM based latent heat storage system within the context of materials, thermal stability and compatibility. Energy Convers. Manag..

[6] Abdelrahman H.E., Wahba M.H., Refaey H.A., Moawad M., Berbish N.S. (2019). Performance enhancement of photovoltaic cells by changing configuration and using PCM (RT35HC) with nanoparticles Al_2_O_3_. Sol. Energy.

[7] Khan Z., Khan Z.A., Sewell P. (2019). Heat transfer evaluation of metal oxides based nano-PCMs for latent heat storage system application. Int. J. Heat Mass Transf..

[8] Salem M.R., Elsayed M.M., Abd-Elaziz A.A., Elshazly K.M. (2019). Performance enhancement of the photovoltaic cells using Al_2_O_3_/PCM mixture and/or water cooling-techniques. Renew. Energy.

[9] Motahar S., Nikkam N., Alemrajabi A.A., Khodabandeh R., Toprak M.S., Muhammed M. (2014). A novel phase change material containing mesoporous silica nanoparticles for thermal storage: A study on thermal conductivity and viscosity. Int. Commun. Heat Mass Transf..

[10] Warzoha R.J., Weigand R.M., Fleischer A.S. (2015). Temperature-dependent thermal properties of a paraffin phase change material embedded with herringbone style graphite nanofibers. Appl. Energy.

[11] Ho C.J., Gao J.Y. (2009). Preparation and thermophysical properties of nanoparticle-in-paraffin emulsion as phase change material. Int. Commun. Heat Mass Transf..

[12] Saydam V., Duan X. (2019). Dispersing different nanoparticles in paraffin wax as enhanced phase change materials. J. Therm. Anal. Calorim..

[13] Nourani M., Hamdami N., Keramat J., Moheb A., Shahedi M. (2016). Thermal behavior of paraffin-nano-Al_2_O_3_ stabilized by sodium stearoyl lactylate as a stable phase change material with high thermal conductivity. Renew. Energy.

[14] Choi D.H., Lee J., Hong H., Kang Y.T. (2014). Thermal conductivity and heat transfer performance enhancement of phase change materials (PCM) containing carbon additives for heat storage application. Int. J. Refrig..

[15] Ilyas S.U., Pendyala R., Marneni N., Korada V., Hisham B., Hamid N. (2017). Stability of Nanofluids. Engineering Applications of Nanotechnology. Topics in Mining, Metallurgy and Materials Engineering.

[16] Che Sidik N.A., Jamil M.M., Japar W.M.A.A., Adamua I.M. (2017). A review on preparation methods, stability and applications of hybrid nanofluids. Renew. Sustain. Energy Rev..

[17] Xian H.W., Che Sidik N.A., Saidur R. (2020). Impact of different surfactants and ultrasonication time on the stability and thermophysical properties of hybrid nanofluids. Int. Commun. Heat Mass Transf..

[18] Li D., Fang W., Feng Y., Geng Q., Song M. (2019). Stability properties of water-based gold and silver nanofluids stabilized by cationic gemini surfactants. J. Taiwan Ins. Chem. Eng..

[19] Zhao M., Lv W., Li Y., Dai C., Zhou H., Song X., Wu Y. (2018). A Study on preparation and stabilizing mechanism of hydrophobic silica nanofluids. Materials.

[20] Yang Y., Luo J., Song G., Liu Y., Tang G. (2014). The experimental exploration of nano-Si_3_N_4_/paraffin on thermal behavior of phase change materials. Thermochim. Acta.

[21] Motahar S., Nikkamb N., Alemrajabi A.A., Khodabandeh R., Toprak M.S., Muhammed M. (2014). Experimental investigation on thermal and rheological properties of n-octadecane with dispersed TiO_2_ nanoparticles. Int. Commun. Heat Mass Transf..

[22] Kumar K.R.S., Dinesha R., Roseline A.A., Kalaiselvam S. (2017). Performance analysis of heat pipe aided NEPCM heat sink for transient electronic cooling. Microelectron. Reliab..

[23] Khodadadi J.M., Hosseinizadeh S.F. (2007). Nanoparticle-enhanced phase change materials (NEPCM) with great potential for improved thermal energy storage. Int. Commun. Heat Mass Transf..

[24] John M.R.W., Mamidi T., Subendran S., Subramanian L.R.G. (2018). Experimental investigation of low-temperature latent heat thermal energy storage system using PCM and NEPCM. IOP Conf. Ser. Mater. Sci. Eng..

[25] Barreneche C., Martín M., Calvo-de la Rosa J., Majó M., Fernández A.I. (2019). Own-synthetize nanoparticles to develop nano-enhanced phase change materials (NEPCM) to improve the energy efficiency in buildings. Molecules.

[26] Martin M., Villalba A., Fernández A.I., Barreneche C. (2019). Development of new nano-enhanced phase change materials (NEPCM) to improve energy efficiency in buildings: Lab-scale characterization. Energy Build..

[27] Zhang X., Zhao X., Smith S., Xu J., Yu X. (2012). Review of R&D progress and practical application of the solar photovoltaic/thermal (PV/T) technologies. Renew. Sustain. Energy Rev..

[28] Bakan G., Gerislioglu B., Dirisaglik F., Jurado Z., Sullivan L., Dana A., Lam C., Gokirmak A., Silva H. (2016). Extracting the temperature distribution on a phase-change memory cell during crystallization. J. Appl. Phys..

[29] Ding F., Yang Y., Bozhevolnyi S.I. (2019). Dynamic metasurfaces using phase-change chalcogenides. Adv. Opt. Mater..

[30] Gerislioglu B., Ahmadivand A. (2019). The role of electron transfer in the nonlinear response of Ge_2_Sb_2_Te_5_-mediated plasmonic dimers. Photonics.

[31] Gerislioglu B., Ahmadivand A., Karabiyik M., Sinha R., Pala N. (2017). Reconfigurable antennae: VO_2_-based reconfigurable antenna platform with addressable microheater matrix. Adv. Electron. Mater..

[32] Aguila B., Vasco D.A., Galvez P., Zapata P.A. (2018). Effect of temperature and CuO-nanoparticle concentration on the thermal conductivity and viscosity of an organic phase-change material. Int. J. Heat Mass Transf..

[33] Bayat M., Faridzadeh M.R., Toghraie D. (2018). Investigation of finned heat sink performance with nano enhanced phase change material (NePCM). Therm. Sci. Eng. Prog..

[34] Vajjha R.S., Das D.K., Namburu P.K. (2010). Numerical study of fluid dynamic and heat transfer performance of Al_2_O_3_ and CuO nanofluids in the flat tubes of a radiator. Int. J. Heat Fluid Flow.

[35] Bondareva N.S., Buonomo B., Manca O., Sheremet M.A. (2019). Performance of the finned nano-enhanced phase change material system under the inclination influence. Int. J. Heat Mass Transf..

[36] Bondareva N.S., Sheremet M.A. (2018). Numerical investigation of the two-dimensional natural convection inside the system based on phase change material with a source of volumetric heat generation. Thermophys. Aeromech..

[37] Yang Y.-T., Wang Y.-H. (2012). Numerical simulation of three-dimensional transient cooling application on a portable electronic device using phase change material. Int. J. Therm. Sci..

[38] Bondareva N.S., Sheremet M.A. (2018). Conjugate heat transfer in the PCM-based heat storage system with finned copper profile: Application in electronics cooling. Int. J. Heat Mass Transf..

[39] Bondareva N.S., Sheremet M.A. (2016). Effect of inclined magnetic field on natural convection melting in a square cavity with a local heat source. J. Magn. Magn. Mater..

[40] Gau C., Viskanta R. (1986). Melting and solidification of a pure metal on a vertical wall. J. Heat Transf..

[41] Gong Z.X., Mujumdar A.S. (1997). Flow and heat transfer in convection-dominated melting in a rectangular cavity heated from below. Int. J. Heat Mass Transf..

[42] Casano G., Piva S. (2014). Experimental and numerical investigation of a phase change energy storage system. J. Phys. Conf. Ser..

[43] Vajjha R.S., Das D.K. (2009). Experimental determination of thermal conductivity of three nanofluids and development of new correlations. Int. J. Heat Mass Transf..

